# Contribution of Regulatory T Cell Methylation Modifications to the Pathogenesis of Allergic Airway Diseases

**DOI:** 10.1155/2021/5590217

**Published:** 2021-06-19

**Authors:** Jiani Li, Jichao Sha, Liwei Sun, Dongdong Zhu, Cuida Meng

**Affiliations:** ^1^Department of Otolaryngology Head and Neck Surgery, China-Japan Union Hospital of Jilin University, Changchun, China; ^2^Jilin Provincial Key Laboratory of Precise Diagnosis and Treatment of Upper Airway Allergic Diseases, China

## Abstract

Regulatory T (Treg) cells are a subtype of CD4^+^ T cells that play a significant role in the protection from autoimmunity and the maintenance of immune tolerance via immune regulation. Epigenetic modifications of Treg cells (i.e., cytosine methylation at the promoter region of the transcription factor, Forkhead Box P3) have been found to be closely associated with allergic diseases, including allergic rhinitis, asthma, and food allergies. In this study, we highlighted the recent evidence on the contribution of epigenetic modifications in Treg cells to the pathogenesis of allergic diseases. Moreover, we also discussed directions for future clinical treatment approaches, with a particular emphasis on Treg cell-targeted therapies for allergic disorders.

## 1. Introduction

Allergic diseases constitute a group of chronic inflammatory diseases that include allergic bronchial asthma, allergic rhinitis (AR), allergic conjunctivitis, and food allergies. These disorders are commonly known as atopic diseases, characterized by extensive clinical manifestations that are primarily mediated by immunoglobulin E (IgE). Over the past few decades, the prevalence and severity of these IgE-mediated allergic diseases have increased dramatically and are becoming a major global health concern [1].

Allergic diseases arise due to an unbalanced immune response with allergic inflammation that is mediated by T helper 2 (Th2) lymphocytes. A Th2-type immune response is characterized by the release of a wide variety of cytokines, including IL-4, IL-5, IL-13, and IL-9. These cytokines mediate the effector phase of an allergic reaction and contribute to the inflammatory response [1]. There is a genetic component to allergic diseases with related gene products that regulate and/or participate in the proliferation, activation, and presentation of allergens to T and B lymphocytes.

Increased exposure to a comprehensive list of environmental risk factors can contribute to the development of asthma and other allergic disorders. These factors include tobacco smoke, allergen exposure, SO_2_, and NO_2_ pollution. In contrast, the absence of exposure to some protective environmental factors (e.g., farm microorganisms or traditional diets) has also been statistically linked to an increased risk of asthma and allergies [2].

The effects of environmental factors on allergic disorders are thought to be mediated, at least in part, by epigenetic modifications (e.g., DNA methylations and/or histone modifications). DNA methylation is a biochemical process by which the methyl group is added to cysteine or adenine bases in DNA and is considered to be a well-established epigenetic modification that silences genes. The level of DNA methylation is dynamically regulated throughout the entire lifespan, and an aberrant alteration of DNA methylation has been associated with allergic disease [3]. For example, DNA hypomethylation of the *IL-13* gene was shown to enhance the production of IL-13, which led to an IgE overload and increased AR to house dust mite (HDM) [4]. Moreover, in a cohort study of 455 children, it was found that reproducible nasal brush DNA methylation was associated with asthma and rhinitis [5]. In addition, environmental factors, such as active/secondhand smoke, exposure to pets, household dampness, and molds, could affect nasal epithelial DNA methylation, which contributes to asthma and rhinitis. In addition, DNA methylation plays a significant role in the growth, activation, and stability of T cell effector function. For instance, demethylation of the Forkhead box protein P3 (Foxp3) is conducive to the development of regulatory T (Treg) cells [6], which can maintain immune tolerance and regulate immunity.

Despite scientific advances, the reason for the sharp increase in allergic disease severity and morbidity over the past 70 years remains largely unknown. In this study, we highlighted the role of epigenetic modifications (i.e., DNA methylation) of Treg cells in the pathogenesis of allergic disorders. Furthermore, the potential use of directed epigenetic therapy as a novel antiallergic treatment was discussed.

## 2. Status of Treg Cells in Allergic Diseases

Allergic diseases are classified as chronic inflammatory disorders that fail to elicit an effective tolerant immune response to allergens. Tolerance to allergens primarily depends on allergen-specific Treg cells that mediate a persistent nonresponsiveness to an offending allergen [7]. Based on their source, Treg cells can be categorized into two main types: natural Treg (nTreg) cells that have a thymic origin and induced Treg (iTreg) cells located within the periphery. Functionally, nTreg cells mediate tolerance to self-antigens, whereas iTreg cells mainly control the immune response to non-self-antigens through the production of transforming growth factor-*β* (TGF-*β*) [8]. Both of these cell types are independent in their generation and are functionally complementary in the promotion of peripheral tolerance [9].

**Figure 1:**
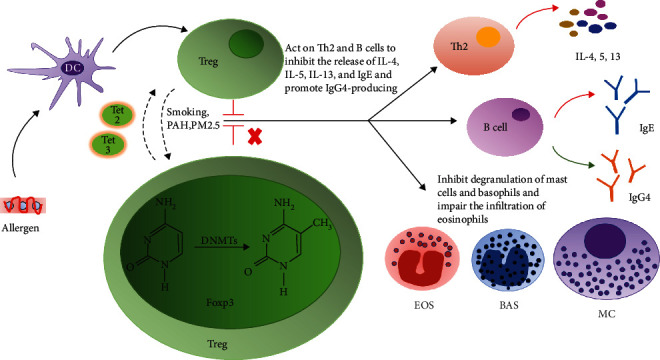
The role of Treg cells in the allergic response and the influence of DNA methylation. Treg cells inhibit a multitude of allergic reactions induced by allergens. Treg cells can act on Th2 and B cells to block the release of inflammatory cytokines (e.g., IL-4, IL-5, and IL-13), IgE, and small molecules. Treg cells can also suppress mast cell and basophil degranulation and tissue infiltration by eosinophils, as well as promote the synthesis and release of IgG4 to relieve the immune response. Following exposure to smoke, PAH, PM2.5, and other factors, DNA methylation of Treg cells is modified, which alters the effect of Treg cells on related immune cells and the allergic response. Finally, Tet2 and Tet3 work together to maintain demethylation of the Foxp3 gene in Treg cells, ensuring the stability of function.

Although allergic diseases are associated with genetic predisposition, there is evidence that the immune response is directly involved in disease development. To initiate an allergic response, Th2 cells, group 2 innate lymphoid cells (ILC2), Th2 cytokines (e.g., IL-4, IL-5, and IL-13), and IgE work together to produce the immediate symptoms of allergic disease. Functionally, Treg cells both balance the immune response to antigens and inhibit the inflammatory response, thereby reducing the symptom severity (Figure 1). Furthermore, Treg cells inhibit Th2 and ILC2 cell activation, decreasing the secretion of IL-4, IL-5, IL-13, and other inflammatory factors [9]. Treg cells also induce the synthesis of allergen-specific IgG4 by B cells. The inhibition of IgE synthesis and reduced activation of antigen-specific T cells can alleviate allergic reactions. Treg cells can also directly or indirectly inhibit mast cell and basophil degranulation, as well as impair the infiltration of eosinophils and effector T cells into inflammatory sites [10]. Given the crucial role of Treg cells in the immune response, an insufficient number or malfunction of Treg cells can aggravate allergic symptoms (Figure 1).

It is acknowledged that Foxp3 is the master transcription factor for the proliferation and differentiation of Treg cells, which functions as an important molecular component of Treg cells. Functional Treg cells are impaired in Foxp3− mice in which allergic reactions are not limited, exhibiting a rapid increase in IgE and eosinophils, as well as imbalanced Th1 and Th2 cytokines with uncontrolled secretion, such as IL-4, IL-5, and IL-13. [11]. In two separate studies, Treg cell depletion (DEREG) in mice caused a deficiency in oral tolerance [12] and was also found to acutely enhance increased T cell infiltration, resulting in colonic inflammation due to an increase in Firmicutes within the gut microbiota [13]. Moreover, mutations in Foxp3 led to dysregulated immune development, multiple endocrine adenosis, bowel disease, and X-linked syndrome. In addition to causing autoimmune diseases, Foxp3 dysregulation could also result in typical allergic inflammation in humans from infancy through to adulthood [14]. In summary, Treg cells play an important role in allergic diseases, as well as in inflammation and autoimmune diseases with genetic, physiological, and immunologic origins. Epigenetic modifications are essential to Treg function and the stability of Foxp3 expression.

## 3. Mechanisms and Significance of Treg Methylation

### 3.1. Structure of Foxp3 and Ten-Eleven Translocation Proteins (Tets)

**Figure 2:**
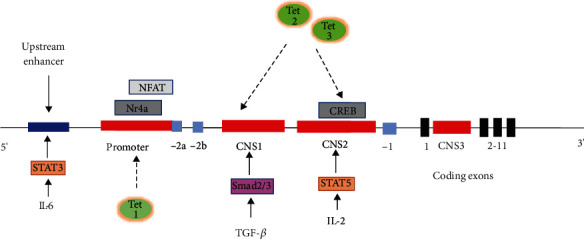
The structure of the Foxp3 gene. Red bars represent four important cis-regulatory elements of the Foxp3 gene. Light-blue and black bars represent three noncoding and 11 coding exons. Of the three enhancers, CNS2 is important for stable Foxp3 expression. Cytokines (e.g., IL-2, IL-6, and TGF-*β*) can act on related regions to regulate Foxp3 expression. Among the three Tet enzymes, Tet1 acts on promoters and transcription start sites. Tet2 mainly acts on gene bodies, whereas Tet2 and Tet3 work together to regulate CNS2 and CNS3 demethylation, as well as other regulatory regions.

Epigenetics is defined as heritable changes in gene expression without changes in the genomic DNA sequence [15]. Such changes include modifications in DNA bases and histones, as well as chromatin remodeling. Base modifications include primarily methylation/hydroxylmethylation demethylation of cytosine and adenosine. DNA methylation refers to the addition of a methyl group to cytosine or adenosine generating 5-methylcytosine (5mC), which is catalyzed by DNA methyltransferases (DNMTs) [16]. There are three types of DNMTs (DNMT1, DNMT3a, and DNMT3b); each of which play a unique role in DNA methylation. DNMT1 is the main DNMT and is important for the maintenance of a gene's DNA methylation status. DNMT3a and DNMT3b are responsible for de novo methylation and mediate methylation-independent gene repression. In somatic cells, this modification is typically found in dinucleotide CpG islands [17]. In Treg cells, Foxp3 functions as the main developmental regulator, with its constitutive expression necessary for the maintenance of normal Treg-suppressive function. Structurally, the Foxp3 promoter and three enhancers are the important cis-regulatory elements for Foxp3 expression (Figure 2) [18]. The promoter region of Foxp3 contains significant transcription factor binding sites for activator protein-1 (AP-1), nuclear factor of activated T cells (NFAT), and nuclear receptor 4a (NR4a). Several intronic enhancers must be activated and looped to the Foxp3 promoter in order to regulate Foxp3 expression in a stable and precise manner. In particular, Foxp3 harbors three conserved enhancers, essentially three conserved noncoding sequences (CNS), including CNS1, CNS2, and CNS3, with the numbers reflecting their distance from the transcriptional start site [19]. Of these, the deletion of CNS2 results in a gradual loss of Foxp3 expression through cell division. Further analysis of CNS2-deleted mice revealed that this enhancer determined the Treg cell lineage during inflammatory states [20]. The CNS2 enhancer is continuously hypomethylated in nTreg cells, while partially demethylated in iTreg cells, potentially contributing to a lack of stability [21]. Further demethylation may facilitate transcription factor accessibility. Many transcription factors (e.g., CREB, Runx1, and Foxp3) bind to CNS2 in a demethylation-dependent manner, suggesting 5-mC-conferred interference in transcription factor binding.

A CpG-rich region within the Foxp3 gene locus is known as the Treg-specific demethylated region (TSDR). DNA demethylation typically occurs in this region, which contributes to stable Foxp3 expression. CNS2 represents the main TSDR and is heavily methylated in freshly generated iTregs. Commitment thymic Treg development is induced by stable Foxp3 expression through demethylation of the TSDR, which maintains homeostasis and regulates the inflammatory response [22]. TSDR demethylation is catalyzed by TET enzymes that oxidize 5mC to 5-hydroxymethylcytosine (5hmC), as well as the stepwise oxidation products 5 formyl cytosine (5-fC) and 5-carboxylcytosine (5-caC) [23, 24].

In mammals, there are three Tet paralogs (Tet1, Tet2, and Tet3) that have a catalytic domain at the carboxyl terminus and a distinct tissue-specific and spatial expression pattern. Tet1 is primarily involved in the demethylation of promoters and transcription initiation sites. Tet2 mainly demethylates gene bodies, whereas Tet3 plays an important role in embryonic development [25–27]. Several reports have demonstrated a critical role of Tet enzymes in Treg cell function. For example, Tet2 and Tet3 function together to maintain the demethylation of CNS1, CNS2, and other several regulatory regions of the Foxp3 gene in nTreg cells [17]. The stability of Foxp3 expression is attenuated in the absence of Tet2 and Tet3. Furthermore, IL-2 plays a dual function during the immune response related to the stability of Foxp3^+^ Treg cells. In IL-2-deficient Treg cells, the downregulation of Tet2 demethylation at the TSDR of Foxp3 results in unstable Foxp3 expression. During the development of nTreg cells, IL-2 has been shown to play an important role in Tet2 upregulation, which is essential for stable Foxp3 expression (Figure 2) [23]. In this context, Foxp3 TSDR methylation is inversely correlated with Tet2 and Foxp3 expression; however, the level of Foxp3 expression is proportionate to the percentage of Treg cells [16]. Taken together, Tet family members promote nTreg cell stability and immune homeostasis by regulating Foxp3 epigenetic modifications.

### 3.2. Treg Methylation Modifications in Allergic Airway Diseases

Many common clinical allergic diseases (e.g., AR, asthma, and anaphylactic reactions) are caused by multiple factors (e.g., smoke, food, and a high-fiber diet) that have been pathologically linked to epigenetic modifications (Figure 1). Of the epigenetic modifications, DNA base methylation of Treg cells contributes to the pathogenesis of these allergic diseases.

#### 3.2.1. AR

Immunologically, AR is considered to be a dysregulation of functional CD4^+^ T-cell subpopulations, including a Th1/Th2 immune response imbalance mediated by Th2-mediated hyperactivity and the inhibition of Th1 responses [28]. In addition to a Th1/Th2 imbalance, Treg cells make a key supplemental contribution to AR mechanisms. In AR patients, Treg cell malfunctions induced by small molecules and proteins that downregulate Foxp3 expression significantly contribute to AR pathogenesis. Of these molecules, prostaglandin E2 (PGE2) is the most abundant prostanoid in the human body and can activate E prostanoid receptor 4 (EP4) via activation of the cyclic AMP- (cAMP-) dependent protein kinase A (PKA) pathway in Treg cells. This pathway is known as the PGE2-EP4-cAMP signaling pathway, which pathologically mediates the development of AR through the inhibition of Treg cell differentiation [29]. In addition, miR-155, miR-181a [27], Notch signaling [28], Tangeretin [29], and hydrogen-rich saline (HRS) [30] all regulate Treg cell proliferation and effector responses through regulatory pathways, each of which may represent a potential target for AR therapy.

A significant increase in DNA methylation and an associated decrease in Foxp3 expression in Treg cells were detected in 256 AR or juvenile asthma patients [30]. This observation led to the hypothesis that the epigenetic dysfunction of Treg cells that resulted from inappropriate Foxp3 DNA methylation contributed to AR pathogenesis. Aberrant Foxp3 methylation has been associated with the downregulation of Tet2, which primarily regulated the demethylation of gene bodies. Indeed, Foxp3 mRNA and protein levels were significantly reduced in the Treg cells of AR patients relative to normal controls [16]. Consistently, Tet2 expression was also downregulated in AR, suggesting that Tet2 modulated Treg cell function through the regulation of Foxp3 methylation. Moreover, Tet2 downregulation led to the hypermethylation of the Foxp3 gene, which suppresses Foxp3 expression in the Treg cells of the AR patients.

Epigenetic inheritance of AR from mother to offspring remains controversial. Interestingly, a reversible immune abnormality has been reported in offspring affected by maternal mouse Foxp3 DNA hypermethylation following exposure to *Dermatophagoides pteronyssinus* 1 (Der p1) [31]. In another study, no significant difference was detected in the Foxp3 promoter between AR mouse mothers and their offspring [3]; however, this observation alone did not indicate a conflict with a Foxp3 hypermethylation phenotype that mediates AR. On one hand, many conserved CpG-rich regions are distributed upstream of the enhancer, promoter, and intronic regions of the Foxp3 gene. On the other hand, of the regions of Foxp3 methylation, the methylation status of only one region may fail to adequately reflect the hypermethylation of the Foxp3 promoter in both AR mother mice and their offspring. Furthermore, a limited number of mice in these experiments may have affected the accuracy of the assessment. Thus, it may be appropriate to infer that both the AR maternal mice and their offspring have an abnormal immunological status, which affects Treg cells. Although the epigenome of AR offspring is potentially susceptible to AR, whether the mice develop AR may depend on the exposure to environmental allergens and other immune stimulants during their lifespan [3].

#### 3.2.2. Asthma

Asthma is a type of chronic airway inflammation characterized by increased airway reactivity, airway remodeling, and reversible airway obstruction linked to increased morbidity and healthcare utilization [32]. Recently, there are an increasing number of studies that link the environment factors to epigenetic modifications culminated in asthma. Ambient air pollution (AAP) could impair Treg cell function and aggravate the symptoms of asthma via the upregulation of Foxp3 methylation levels [33]. Further studies indicate a dose-response relationship between the exposure to AAP and Foxp3 methylation levels in children.

It has been well established that common environmental pollutants (e.g., polycyclic aromatic hydrocarbons (PAHs), CO, NO_2_, and PM2.5) are associated with alterations in the differentially methylated regions of Foxp3 [30, 34]. PM2.5 promotes Th17 cell differentiation via the aryl hydrocarbon receptor (AhR) and impairs the differentiation of Treg cells through the inhibition of Tet2 activity, which consequently leads to the hypermethylation of the Foxp3 locus. Thus, PM2.5 can disturb the Treg/Th17 cell balance and aggravate asthma in an AhR-dependent manner [35].

Th17 cells are a subtype of CD4^+^ T cells that release cytokines (e.g., IL-17), which can amplify inflammatory reactions. Compared to the significant role of Foxp3 in Treg cells, the retinoic acid-related orphan receptor-*γ*t (ROR*γ*t) is essential for Th17 development and function [36]. Treg and Th17 cells can antagonize each other but maintain a balance of immunological homeostasis. An imbalance in Treg and Th17 cells contributes to asthma exacerbation, severity, and disease status [37]. Furthermore, LncRNA-MEG3, a noncoding RNA that participates in the pathogenesis of multiple disease states, can inhibit miRNA-17 as a competing endogenous RNA (ceRNA). This competition regulates ROR*γ*t, disrupts the Treg/Th17 balance, and contributes to asthma pathogenesis [32]. The Treg and Th17 cell balance can be affected by passive smoke inhalation, adenosine receptor A2AR, and the programmed cell death-1 (PD-1)/PD-ligand 1 (PD-L1) pathway; all of which can contribute to the severity of asthma in children [38–40]. Of particular interest, mangiferin and a low-dose lipopolysaccharide (LPS) can improve the Treg/Th17 balance in an asthmatic mouse model, which has potential as a clinical target in the therapy of inflammatory diseases and immunologic disorders [41, 42].

In summary, minimizing environmental pollutant exposure and maximizing the Treg and Th17 cell balance may represent a promising strategy for asthma control and potential clinical targets for asthma therapy.

### 3.3. Treg Methylation and Other Factors

Studies have shown that maternal farm exposure, particulate matter exposure from combustion, and a high-fiber diet can affect the number and function of Treg cells, which influences the potential for the development of allergic disease in offspring.

Exposure to a traditional farming environment during childhood typically lowers the risk for the development of respiratory allergic disease later in life. In a study involving 84 pregnant women with agricultural exposure, increased levels and function of Foxp3^+^ cells were found in cord blood [43]. Moreover, the level and function of Treg cells were associated with Foxp3 promoter demethylation in children from mothers with farm milk exposure compared to that of control mothers [44]. Moreover, smoking-induced methylation of the G protein-coupled receptor 15 (GPR15) is related to the development of mucosal immune tolerance through the regulation of Treg cell numbers. This tolerance may be related through DNA methylation, the methylation associated with cigarette smoking, even after cessation [45].

Feeding mice a high-fiber diet produced distinctive gut microbiota that markedly suppressed allergic airway disease [46]. These mice exhibited enhanced number and functionality of Treg cells as a result of the acetylation of the Foxp3 promoter, which was likely due to impaired histone deacetylases 9 (HDAC9) activity [47, 48]. Food allergies have also been linked to the epigenetic modification of Treg cells. Successful oral immunotherapy of patients with a peanut allergy has been associated with enhanced CpG island demethylation of the Foxp3 gene. In the circulatory system of these patients, iTreg cells exhibit improved functionality and stability in the number of iTreg cells with enhanced immune tolerance to peanuts [49]. Additionally, folic acids, fish oil, and obesity have been thought to be involved in epigenetic modifications in patients with allergic diseases; however, only limited information is available, suggesting the importance of further study [6].

## 4. Cytokine Alterations Mediated by Treg DNA Methylation

Numerous cells and inflammatory cytokines (e.g., IL-17, IL-6, IL-10, and TGF-*β*) induced by alterations in the DNA methylation status of Treg cells become involved in the development of allergic diseases. Understanding these DNA alterations, related cellular responses and cytokines will enhance the understanding of the pathological basis of allergic disease and may ultimately be used to develop novel therapeutic strategies.

The two subtypes of Treg cells (iTreg and nTreg) are differentially stable in that iTreg cells are less stable than nTreg cells during inflammatory conditions. The instability of iTreg cells is due to a deficiency in Foxp3 expression, which results in the production of cytokines (e.g., IL-17 and interferon- (IFN-) *γ*), triggered by the methylation status of CNS2 of the Foxp3 gene [50, 51]. Treg-specific Tet2/Tet3-deficient mice develop a fatal lymphoproliferative disease [24]. In these mice, CD4^+^ Foxp3^+^ T cells were expanded in the mesenteric lymph nodes (LNs) and spleen, displaying elevated levels of IL-17-producing cells as well as serum IgG1, IgE, and IgA. In patients with Henoch-Schönlein purpura (HSP) or asthma, methylation-based silencing of the related genes (e.g., Foxp3, suppressor of cytokine signaling 1 (SOCS1), and SOCS3) disturbed the Treg/Th17 cell balance and critically contributed to pathogenesis of related diseases [52].

The inflammatory cytokine, IL-6, has been shown to suppress the development and function of Treg cells by inhibiting Foxp3 expression. In nTreg cells, IL-6 induces the signal transducer and activator of transcription 3- (STAT3-) dependent methylation of a Foxp3 enhancer by DNMT1 (Figure 2) [53]. Moreover, IL-6-STAT3-induced reductions in Foxp3 expression can be antagonized by B lymphocyte-induced maturation protein 1 (Blimp1), a zinc finger protein, and a transcriptional regulator that supports Treg cell specificity and functions at sites of inflammation [54]. In terms of the associated mechanism, Blimp1 was found to negatively regulate IL-6- and STAT3-dependent Dnmt3a expression (rather than DNMT1), thereby restraining CNS2 methylation in the Foxp3 locus and maintaining continuous Foxp3 expression and suppressing inflammation. DNMT1 is thought to maintain the DNA methylation status of a gene, whereas DNMT3 family members are responsible for de novo methylation. Thus, DNMT3a might be more important for the de novo methylation of constitutively hypermethylated CNS2 of Treg cells. Therefore, although the specific mechanism remains to be investigated, the antagonist activities of STAT3 and Blimp1 with DNMT could potentially represent a promising strategy for the clinical treatment of AR.

Elevated levels of Foxp3-TSDR methylation are inversely correlated with IL-10 and TGF-*β* in patients with acute coronary syndrome (ACS) and are associated with an increased risk of adverse outcomes [55]. In a separate study, increased PAH exposure was related to weakened Treg cell function in both asthmatic and nonasthmatic individuals, potentially via an IL-10-independent mechanism [30, 56]. However, the precise role of cytokines in the epigenetic modification of Treg cells remains unknown.

## 5. Gene Targeting Therapy of Treg Cells

A therapeutic strategy for the treatment of autoimmune and allergic disorders may represent modalities that enhance the number and function of Treg cells [57]. Studies have shown that synthetic methylated DNA, CpG oligonucleotides (ODN), promotes Foxp3 expression in human CD4^+^ T cells with TGF-*β* and IL-2, whereas unmethylated CpG ODN does not; however, the precise mechanism remains unknown [57, 58]. The combination of conjugated CpG ODN and Amb a1, a ragweed pollen antigen, may attenuate acute allergic reactions in AR patients by increasing the specific IgG for Amb a1 and decreasing specific IgE for Amb a1 [59]. Therefore, it is essential to identify the *in vitro* Treg-inducing properties of CpG ODN. Based on the results in animal models, translation to clinical application could provide a promising novel therapeutic strategy. A small molecular size, low cost, and easy production of ODN may provide a wide range of clinical applications for the induction of Treg cells with the possibility of becoming a new form of vaccine, thereby becoming a novel therapy for many types of allergic and autoimmune diseases [57].

The related protein and small molecule-based regulation of Treg cell proliferation and differentiation has been demonstrated to have therapeutic effects for allergic disorders and autoimmune diseases. The primary consideration for such therapy is to maintain the stable expression of Foxp3 in Treg cells and a low toxicity. Although DNMT inhibitors can induce Foxp3 expression, toxicity limits their clinical application. To overcome this concern, the experiment has been conducted in the cultured primitive CD4^+^ T cells with a low concentration of DNMT inhibitors in a TGF-*β*-regulated Treg cell environment [15]. After 24 h, the DNMT inhibitor was removed and the T cells were continuously cultured with TGF-*β* in a Treg environment. With these culture conditions, Foxp3 was stably expressed in Treg cells following the demethylation of its promoter. Treg cells generated using this method were more stable than conventional TGF-*β*-induced Treg cells, which were capable of protection from autoimmune colitis and allergic disease.

All-trans retinoic acid (ATRA) has been reported to induce CD4^+^ CD25^+^ Foxp3^+^ Treg cells by triggering demethylation of the Foxp3 promoter. ATRA treatment of systemic sclerosis (SSc) CD4^+^ T cells induces Foxp3 expression with enhanced immunosuppressive functionality [60]. This observation provides the impetus for the potential clinical application of retinoids for SSc therapy.

In addition to DNMT-targeted therapy, targeting Tet enzymes with small molecule activators could enhance the inductive efficacy of Treg cells. In addition, vitamin C has been shown to potentiate Tet activity and increase the stable expression of Foxp3 in iTreg cells via Tet2/3 [17, 61], suggesting a potential clinical application in transplantation and autoimmune diseases.

Although Treg cell-targeted therapies are expected, achievements from the important investigations described above have shed insight into future studies that can potentially lead to the development of novel and effective therapies for allergic diseases.

## 6. Summary

In conclusion, the epigenetic modification of Treg cells, especially DNA methylation, significantly contributes to the pathogenesis of allergic and autoimmune diseases. AR, asthma, food, and smoke allergy are all closely related to Treg DNA methylation. Although the specific pathogenesis involved in these diseases remains to be investigated, Treg cell-targeted therapies may provide a novel approach for the effective treatment of allergic and autoimmune diseases.
